# Cortical circuit dysfunction in a mouse model of alpha-synucleinopathy *in vivo*

**DOI:** 10.1093/braincomms/fcab273

**Published:** 2021-11-15

**Authors:** Sonja Blumenstock, Fanfan Sun, Carolin Klaus, Petar Marinković, Carmelo Sgobio, Lars Paeger, Sabine Liebscher, Jochen Herms

**Affiliations:** 1 German Center for Neurodegenerative Diseases (DZNE), 81377 Munich, Germany; 2 Center for Neuropathology and Prion Research, Ludwig-Maximilians University Munich, 81377 Munich, Germany; 3 Munich Cluster for Systems Neurology (SyNergy), 81377 Munich, Germany; 4 Institute of Clinical Neuroimmunology, Klinikum der Universität München, Ludwig-Maximilians University, 82152 Martinsried, Germany; 5 Biomedical Center, Medical Faculty, Ludwig-Maximilians University Munich, 82152 Martinsried, Germany

**Keywords:** alpha-synuclein, *in vivo* two-photon calcium imaging, awake mice, Parkinson’s disease, seeding

## Abstract

Considerable fluctuations in cognitive performance and eventual dementia are an important characteristic of alpha-synucleinopathies, such as Parkinson’s disease and Lewy Body dementia and are linked to cortical dysfunction. The presence of misfolded and aggregated alpha-synuclein in the cerebral cortex of patients has been suggested to play a crucial role in this process. However, the consequences of a-synuclein accumulation on the function of cortical networks at cellular resolution *in vivo* are largely unknown. Here, we induced robust a-synuclein pathology in the cerebral cortex using the striatal seeding model in wild-type mice. Nine months after a single intrastriatal injection of a-synuclein preformed fibrils, we observed profound alterations of the function of layer 2/3 cortical neurons in somatosensory cortex by *in vivo* two-photon calcium imaging in awake mice. We detected increased spontaneous activity levels, an enhanced response to whisking and increased synchrony. Stereological analyses revealed a reduction in glutamic acid decarboxylase 67-positive inhibitory neurons in the somatosensory cortex of mice injected with preformed fibrils. Importantly, these findings point to a disturbed excitation/inhibition balance as a relevant driver of circuit dysfunction, potentially underlying cognitive changes in alpha-synucleinopathies.

## Teaser

Blumenstock et al. report brain state-dependent hyperreactivity in somatosensory cortex months after striatal seeding of alpha-synuclein preformed fibrils. A concerted reduction of GAD67 positive interneurons argues for excitation/inhibition imbalance as a driver of cortical network dysfunction.

## Graphical Abstract

**Graphical Abstract:**
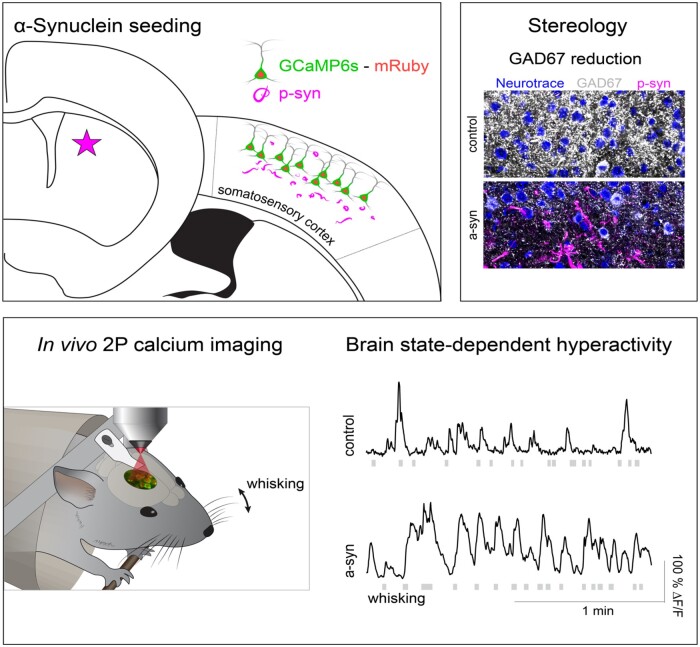

## Introduction

Alpha-synucleinopathies, including Parkinson’s disease, are not only characterized by progressive worsening of motor symptoms, but also by cognitive deficits, which can strongly fluctuate over short time scales. The mechanisms driving these cognitive disturbances, however, remain poorly understood.^1^ In addition to the well-known degeneration within the basal ganglia, there is ample evidence for a pathophysiological involvement of cortical areas linking alpha-synucleinopathies and cognitive decline.^2–4^ At the molecular and cellular level, a link between misfolding, self-aggregation and deposition of the native protein a-synuclein (a-syn) and intraneuronal impairments has been established. Amongst others, it has been shown that pathological conformers of a-syn cause various dysfunctions in, for example endoplasmic reticulum-to-Golgi trafficking, cytoskeleton dynamics, protein degradation, synaptic vesicle cycle and calcium homeostasis.^5^^,^^6^ Importantly, a-syn spreads along anatomically connected structures,^7^ which can be initiated by various molecular forms of a-syn, such as monomers, oligomers or fibrils. When applied acutely in cell culture, preformed recombinant a-syn fibrils (PFFs) can compromise neuronal excitability, trigger intracellular a-syn pathology and cause a rapid loss of spines.^8^ In our previous study, we revealed that striatal seeding of PFFs, results in the loss of spines on pyramidal neurons in the anatomically connected somatosensory cortex (S1) *in vivo*.^9^ However, the underlying relationship between local a-syn aggregation, following its templated misfolding, and neuronal network dysfunction *in vivo* remains unknown. Therefore, we here address the question of how a-syn is affecting cortical circuit function in a mouse model of a-synucleinopathy. To this end, we conduct *in vivo* two-photon calcium imaging in S1 of awake mice 9 months after a unilateral striatal infusion of PFFs and performed stereological analyses to quantify the number of excitatory and inhibitory neurons in the same area.

## Material and methods

### Animals

Wild-type (WT) C57BL/6 mice (Jackson Laboratory) were housed in groups, with food and water provided ad libitum (12/12 h light/dark cycle). Mice of both sexes were used (control: four females, three males, a-syn: six females, 1 male). After cranial window implantation, mice were housed separately. All experiments were approved by the Bavarian government (Az. 55.2-1-54-2532-163-13).

### PFF purification and seeding

Recombinant WT mouse a-syn was purified as described.^10^^,^^11^ PFFs were assembled from purified a-syn monomer (5 mg/ml) by incubation at 37°C and 1400 rpm for 96 h and stored at −80°C.^12^ Directly before injection, PFFs were sonicated (SonoPuls Mini 20). Two-month-old mice were anesthetized with ketamine/xylazine (0.13/0.01 mg/g) and stereotactically injected with 5 µl (25 µg) of PFFs into the dorsal striatum (coordinates relative to the Bregma: +0.2 mm anterior, +2.0 mm from midline, +2.6 mm beneath the dura) of the right hemisphere. Control animals received 5 µl sterile PBS. Injection rate was 300 nl/min for both groups.

### Virus injection and cranial window implantation

Eight months after PFF seeding, a cranial window was implanted as reported.^9^ In short, mice were anesthetized (ketamine/xylazine) and received dexamethasone (0.01 mg/g intraperitoneally) right before surgery. A 4 mm in diameter piece of the skull over the right hemisphere (centre of window: ∼1 mm caudal from bregma, 3 mm lateral from midline) was removed, using a dental drill. AAV2/1.hSyn1.mRuby2.GSG.P2A.GCaMP6s.WPRE.SV4^13^ was injected into 3–4 locations within the cranial window (300 nl/spot, 30 nl/min, 0.2 mm beneath the dura, virus titre ∼10^12^ GC/ml) and the craniotomy sealed with a coverslip using dental acrylic. A head-bar was glued next to the coverslip to allow repositioning of the mouse during imaging. After surgery, mice received Carprophen (5 mg/kg, s.c.) and Cefotaxim (0.06 mg/kg).

### *In vivo* imaging

Four weeks after window implantation, imaging was performed in awake, head-fixed mice,^14^ while they sat still in a restrainer. Neuronal activity in layer 2/3 of S1 (depth 200–300 µm) was probed, using a La Vision Trim Scope [La Vision BioTec GmbH, at 10 Hz frame rates, field of view (FOV) of 220×220 µm at 223 pixel resolution] equipped with a Ti: sapphire two-photon laser (Mai Tai, Spectra Physics), tuned to 940 nm to simultaneously excite mRuby2 and GCaMP6s and emitted light was split at 560 nm and green light (495–560 nm, band pass filter) and red light (>560 nm) were detected by photomultiplier tubes. Mouse behaviour was recorded by a web-camera (pco.pixelfly USB camera) controlled by La Vison Imspector software to synchronize it with the imaging data acquisition. Neuronal activity was also investigated under isoflurane anaesthesia (0.5–1 vol%). Body temperature was kept stable with a heating pad, arterial saturation, breathing and heart rate was monitored by a pulse oximeter.

### Data processing and analysis

Collected images were analysed using custom-written codes in MATLAB and ImageJ. Based on whisker movement, neuronal activity was investigated separately during ‘whisking’ and ‘stationary’ epochs. Two-photon imaging data were full-frame registered, to correct for slight brain displacement, based on the mRuby2 channel. Regions of interest (ROIs) were manually identified using custom-written software in MATLAB and GCaMP6s fluorescence was constructed by averaging the pixel values within the ROI for each imaging frame. Time series were corrected for contamination by local neuropil fluorescence.^15^

Traces were low pass filtered at 5 Hz and slow fluctuations removed by subtracting the eighth percentile within a sliding window of 1000 frames. To estimate F0, we subtracted the 8th percentile in a sliding window of 1 s and used the median of all values below the 70th percentile of this ‘noise band’ as F0. ROIs were classified as active, if Δ*F/F* exceeded 3× of the standard deviation (SD) of the noise band for at least 10 frames (1 s). Whisking epochs were identified by changes in pixel intensities within a ROI positioned over the whisker area in the videorecording of the mouse’s snout. Average pixel intensities within this whisker ROI were corrected for slow fluctuations by subtracting the eighth percentile in a sliding window of 50 frames. Pixel intensities exceeding 3× the SD of the noise band were classified as whisking events. Whisking-associated neuronal activity was assessed by considering transients occurring within a window of 1 s before whisking onset up to 2 s after whisking offset. All other transients were considered spontaneous activity.

Transient kinetics were computed on selected isolated individual calcium transients, which were not followed by a subsequent transient within a time window of 10 s. We computed the first derivative of the calcium trace smoothed over seven frames and identified transients characterized by a first derivative exceeding 3.5× the SD of the baseline.^16^ For each ROI, all selected transients were averaged and normalized to the average intensity within 1 s prior to the transient onset. The resulting peak amplitude, the decay time (i.e. the time needed to drop to 36.8% of the peak amplitude) as well as the half decay time (i.e. the time needed to drop to 50% of the peak amplitude) were computed for each ROI.

The correlation of neuronal activity for all pairs of active neurons in each field of view was assessed by computing the Pearson correlation coefficient R. To this end, traces were smoothed over 25 frames and values lower than 2× SD of the noise band were set to 0. The correlation of activity during whisking and stationary epochs was analysed separately and compared to pairwise correlations derived from shuffled data that were generated by circularly shifting each trace at a random value between 1 to the length of the activity trace. In total, we recorded from 1561 neurons in 32 experiments (field of views) from 7 control mice and 1534 neurons in 32 experiments from 7 a-syn mice.

### Immunohistochemistry

After imaging, mice (control, *n* = 5, a-syn, *n* = 5) were transcardially perfused (4% paraformaldehyde), brains post-fixed in 4% paraformaldehyde overnight and cut into coronal sections (50 μm) on a vibratome. For subsequent stereological analyses, floating sections were stained using anti-Alpha-synuclein-phospho S129 (rabbit polyclonal, Abcam) and anti-glutamic acid decarboxylase 67 (GAD67) (mouse monoclonal, Millipore) antibodies, at 1:1000 dilution, for 48 h at 4°C. Secondary antibodies (1:1000; goat anti-rabbit Alexa 488, goat anti-mouse Alexa 647, Invitrogen) were incubated overnight at 4°C, followed by an incubation with NeuroTrace 530/615 (1:500, ThermoFisher). To quantify synaptic densities, sections were incubated in blocking solution (10% normal goat serum, 10% normal donkey serum, 3% bovine serum albumin, 2% Triton-X in PBS) for 3 h at room temperature (RT) and thereafter incubated in the primary antibody solution, containing antibodies against HOMER-1 (1:500, Synaptic Systems, USA) and Synaptophysin (1:500, Synaptic Systems, USA) for 72 h at 4°C on a horizontal shaker. Sections were washed in PBS (3 × 10 min) and incubated with secondary antibodies for 1.5 h at RT [Donkey anti-Rabbit Alexa Fluor 647 (1:500, Invitrogen, USA) and Goat anti-Mouse Alexa Fluor Plus 405 (1:500, Invitrogen, USA)] chosen to avoid crosstalk with preserved endogenous fluorescence of GCaMP6s and mRuby2. After washing in PBS (3 × 10 min), sections were mounted in mounting medium (ROTHI-Mount FluorCare, Roth, Karlsruhe) and coverslipped.

Images were acquired on a Zeiss LSM900 confocal microscope (Carl Zeiss, Oberkochen) using a 2.5× objective (Plan-Apochromat) for tile scans and a 63× objective (C-Apochromat, oil) for imaging synaptic punctae (three-dimensional 8-bit stacks of 101.41 × 101.41 µM × 2 µm). The pinhole was matched to an optical thickness of 0.5 µm for both wavelengths. For image quantification, the first and sixth plane of each slice was selected and the The SynapseCounter plugin was used in ImageJ/FIJI (Version v1.53j) to analyse colocalization and subsequent identification of synapses. Each parameter was manually checked for fidelity of image processing on the raw images before processing in the plugin (rolling ball radius: 30 px, maximum filter radium 0.5 px, method for threshold adjustment: Otsu, min. synaptic particle size: 1 px^2^, max. synaptic particle size: 300 px^2^). Results from 2 sections per animal were averaged and reported as mean ± SEM. A two-tailed unpaired *t*-test (α = 0.05) was carried out to compare between groups.

### Stereology

Brain sections were scanned on a Zeiss fluorescent microscope (Imager.M2, ZEISS) and analysed using StereoInvestigator^®^ (MBF Bioscience). Seven serial coronal sections spanning the entire hemisphere in the coronal plane, spaced by 600 µm were analysed. Outlines delineating S1 and fiduciary markers were drawn at 2.5× magnification (EC-Plan-NEOFLUAR 2.5X/0.075, ZEISS) using the Neurotrace stain to delineate reference points. Limits for areas of interest were drawn in accordance with the mouse brain Atlas.^17^ The drawn outlines were used to compute the volume of S1. The investigator was blind to genotype and treatment. Cell analysis was performed at 63× magnification (Plan/APOCHROMAT 63X/1.4 Oil DIC, ZEISS), using a 3D counting frame in a sampling grid (Supplementary Table 1). The coefficient of error (Gundersen), *m* = 1, and the average cell counts per sampling site are described for each marker and region (Supplementary Table 1).

### Statistical analysis

We employed student’s *t*-test to compare the average of normally distributed data (e.g. population response to whisking). For non-normally distributed data, we used the ranksum (Mann–Whitney U) test (e.g. fraction of whisking responsive neurons). Distribution of data was compared using the Kolmogorov–Smirnov test (e.g. distribution of frequencies, amplitudes, correlation coefficients). Stereology results were compared using a two-way ANOVA followed by Bonferroni’s *posthoc* test.

### Availability of data and material

The datasets used and/or analysed during the current study are available from the corresponding author on reasonable request.

## Results

### Injection of a-syn PFFs induces formation of Lewy-neurite like aggregates in cortex of WT mice

The injection of a-syn PFFs into the dorsal striatum triggered the formation of intracellular Lewy-neurite like aggregates in remotely connected areas in the brain, including S1 (Supplementary Fig. 1). Intracellular phosphorylated largely fibrillary a-syn aggregates^9^ were found across all layers in S1 9 months after the injection of PFFs, with the highest density in infragranular layers, which contain extensive projections to the dorsal striatum (Supplementary Fig. 1A and B). As described previously by others and our group,^9^^,^^18^^,^^19^ Lewy body-like and Lewy neurite-like inclusions were observed in all a-syn PFF-injected animals (Supplementary Fig. 1C–E).

### Hyperreactivity in somatosensory cortex in a-syn PFF injected mice

**Figure 1:**
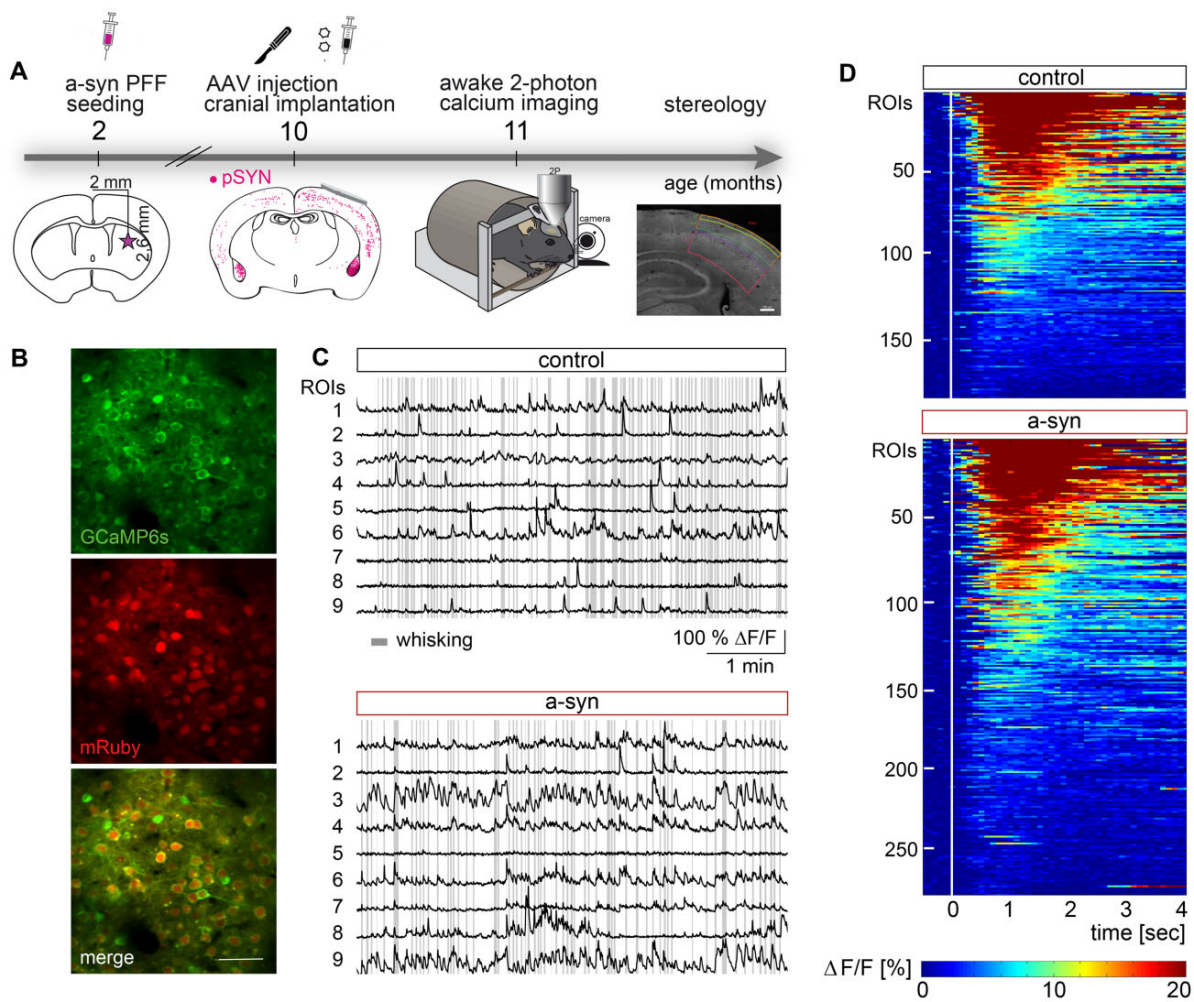
**Probing neuronal function in S1 of behaving mice upon striatal injection of a-syn PFFs.** (**A**) Timeline of experiments. Mice received a striatal injection of PFFs at the age of 2 months, followed by the injection of AAV2/1.hSyn1.mRuby2.P2A.GCaMP6s into the somatosensory cortex (S1) and the implantation of a cranial window 8 months later when PFFs are globally present [assessed by immunofluorescence of phospho-synuclein (pSYN)]. One month later *in vivo* imaging experiments were conducted, after which mice were sacrificed and stereology was performed. (**B**) Representative example of a FOV. (**C**) Calcium traces of individual ROIs are shown for control and a-syn mice and referenced by whisking epochs (grey lines). (**D**) Heat maps depicting the average neural response to whisking onset (white line, normalized to the average activity within 0.5 s before whisking onset) for whisking responsive cells (control: 187 of 1561 neurons, a-syn: 276 of 1534 neurons). Scale bar in B 50 µm.

To assess, whether a-syn accumulation in S1 results in cortical dysfunction, we performed *in vivo* two-photon Ca^2+^ imaging in awake mice 9 months after PFF-seeding (Fig. 1A–C). Using the pan-neuronal promoter hsyn1 to express GCaMP6s and mRuby2 primarily resulted in the transduction of excitatory neurons. In total, only ∼4% of all GCaMP expressing neurons were inhibitory (identified by the expression of GAD67, Supplementary Fig. 2), which is in line with previous reports.^15^^,^^20^ To characterize neuronal function, we quantified neuronal activity levels during stationary/quiescent epochs as well in response to whisking (Fig. 1D).

**Figure 2:**
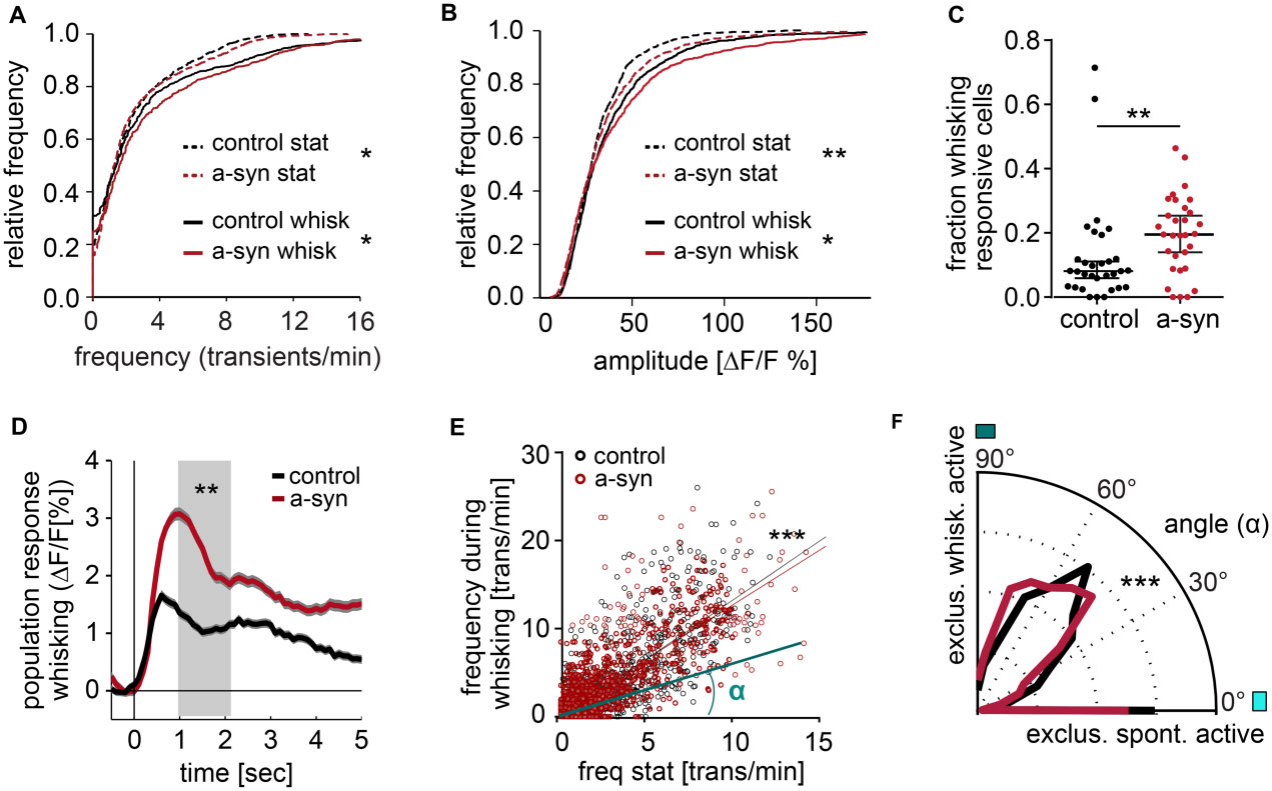
**Neuronal hyperreactivity in S1 upon striatal injection of a-syn PFFs.** (**A**) Calcium transient frequency in S1 is increased in a-syn mice both during quiescence (stat) as well as during whisking-associated epochs [stationary epochs, frequency *P* = 0.016, whisking-associated frequency *P* = 0.011, both Kolmogorov–Smirnov (KS) test]. (**B**) Transient amplitudes during quiescence (stat) and during whisking-associated epochs are larger in a-syn mice (stationary epochs, amplitudes *P* = 0.0019, amplitudes during whisking, *P* = 0.02, both KS test, control *n* = 1561 ROIs, a-syn *n* = 1534 ROIs). (**C**) Fraction of whisking responsive neurons in each experiment (*P* = 0.003, ranksum test, control *n* = 32 experiments, a-syn *n* = 32 experiments). (**D**) Population response of neurons to whisking onset (control: 1561 neurons, a-syn 1534 neurons). (**E**) Relationship of the stationary neuronal frequency and the activity associated with whisking in control (black, *n* = 1561 neurons) and a-syn mice (red, *n* = 1534 neurons, control *R*^2^ = 0.63, *y* = 1.36*x* − 0.07; *P* < 0.0001; a-syn *R*^2^ = 0.63, *y* = 1.26*x* + 0.44, *P* < 0.0001). To compare state-specific neuronal activity levels, the angle α was computed for each neuron, as exemplified for one neuron (light blue line and angle). (**F**) The distribution of these angles is significantly different in a-syn mice, with more neurons favouring activity during whisking epochs [angle of 0° indicates neuronal activity exclusively during stationary (quiescent) epochs, while 90° would indicate exclusive whisking-associated neuronal activity, *P* < 10^−4^, KS test]. Data are mean ± standard error of the mean (SEM). **P < 0.01, ***P < 0.001

In a-syn mice, neuronal activity was increased both during stationary and whisking epochs as evidenced by an increase in transient frequency (Fig. 2A) as well as an increase in calcium transient amplitudes in awake a-syn mice (Fig. 2B), an effect that was not observed under anesthetized conditions (Supplementary Fig. 3A–C).

As behaviour-associated responses rely on the proper integration of synaptic inputs and their regulation via inhibitory connections, this analysis can be instrumental in unravelling excitatory/inhibitory (E/I) imbalances within microcircuits. We chose to investigate the whisking-associated signal as whisking had been shown to trigger both sensory and non-sensory responses in S1 neurons, even in the absence of touch.^21^ Moreover, whisking is associated with a change in brain state compared to quiet wakefulness.^22^ In our experimental setup mice were headfixed and precluded from touching any surfaces with their whiskers, we hence did not record direct sensory-evoked signals in S1.

When comparing the neuronal response to whisking, we found a strong increase in a-syn PFF seeded mice (Fig. 1D), both at the level of individual neurons as well as on the population level (Figs 1D, 2C and D). More specifically, the fraction of neurons responsive to whisking (i.e. neurons that display a significant increase in Δ*F/F* within 0.5–1.5 s after each whisking onset compared to their activity within 0.5 s prior to whisking onset) was increased in a-syn mice (Fig. 2C). In addition, the average response of each active neuron to individual onsets of whisking was also significantly increased in a-syn mice (Fig. 2D). Importantly, both effects reached significance at the level of individual mice (Supplementary Fig. 4F and G) and correlated with the level of a-syn load (Supplementary Fig. 4H). We next asked whether spontaneously active neurons, are more active during whisking, indicative of a rather general increase in excitability. Indeed, the level of spontaneous activity strongly correlated with the whisking-associated activity in both control and a-syn mice (Fig. 2E). To characterize the relationship between spontaneous and whisking-associated neuronal activity for each cell, we computed the angle between whisking-associated versus spontaneous transient frequency (Fig. 2E). This analysis revealed that the relationship between spontaneous and whisking-associated activity was more broadly distributed in a-syn mice compared to control mice (Fig. 2F), with a larger fraction of neurons favouring activity during whisking over spontaneous activity in a-syn mice. Importantly, overall whisking behaviour and pattern did not differ between control and a-syn mice (Supplementary Fig. 4A–E), excluding that behavioural differences could account for changes in whisking-associated neuronal activity.

**Figure 3:**
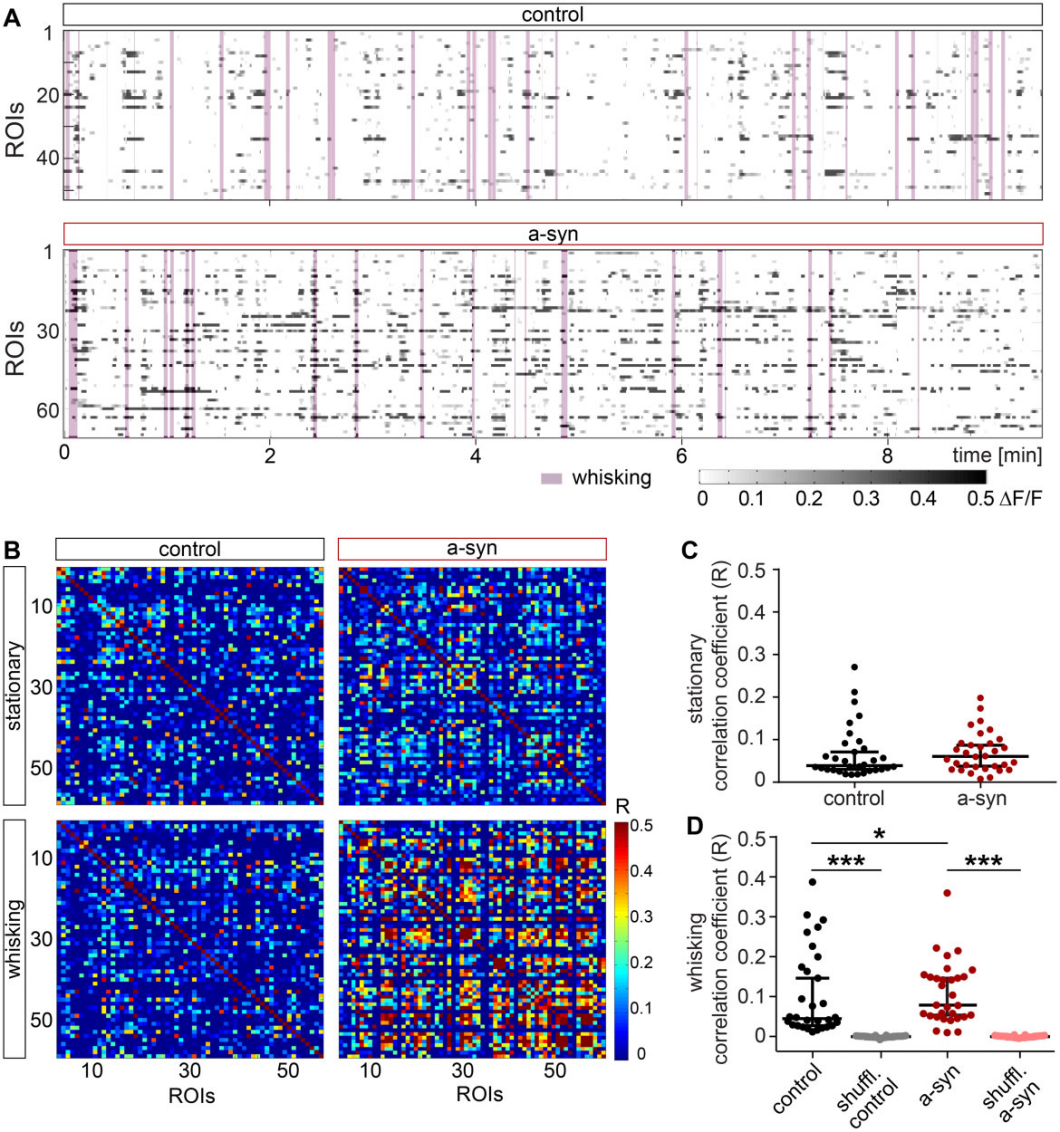
**Striatal PFF seeding elevates pairwise neuronal correlations during whisking in S1.** (**A**) Example raster plots depicting activity of each ROI within an FOV referenced by whisking (purple area) in control and a-syn mice. (**B**) Correlograms of pairwise correlations during stationary and whisking-associated epochs in a control and an a-syn mouse. (**C**) Average pairwise correlations of individual experiments did not differ (*P* = 0.43, KS test, control *n* = 32 experiments, a-syn *n* = 32 experiments), while (**D**) whisking-associated correlations were significantly increased in a-syn compared to control mice and each to shuffled data (control versus a-syn *P* = 0.026, control versus shuffled control *P* < 0.0001, a-syn versus shuffled a-syn *P* < 0.0001, KS test, control *n* = 32 experiments, a-syn *n* = 32 experiments). Data are individual experiments superimposed by the median ± 95% confidence interval (**C** and **D**). * *P* < 0.05, *** *P* < 0.001.

We next addressed whether the kinetics of single calcium transients differ between neurons in control and a-syn mice. We identified individual calcium transients, which were not followed by another transient within the subsequent 10 s and compared the decay (τ), and the half decay time of the mean transient of each ROI (Supplementary Fig. 5A and B). While, finding a significant increase in the Ca^2+^ transient amplitude in a-syn mice (Supplementary Fig. 5C), the kinetics remained unaltered during both wakefulness (Supplementary Fig. 5D and E) and under anaesthesia (Supplementary Fig. 5F–I). As alterations in the delicate balance of excitation and inhibition can affect the degree of synchrony of neuronal activity, we next analysed the correlation between all pairs of active neurons within a field of view. We did not find any difference during stationary epochs between the groups (Fig. 3A–C). However, whisking-associated correlation of neuronal activity was increased (Fig. 3D) in a-syn PFF seeded mice.

### Stereological analysis of neuronal densities

**Figure 4:**
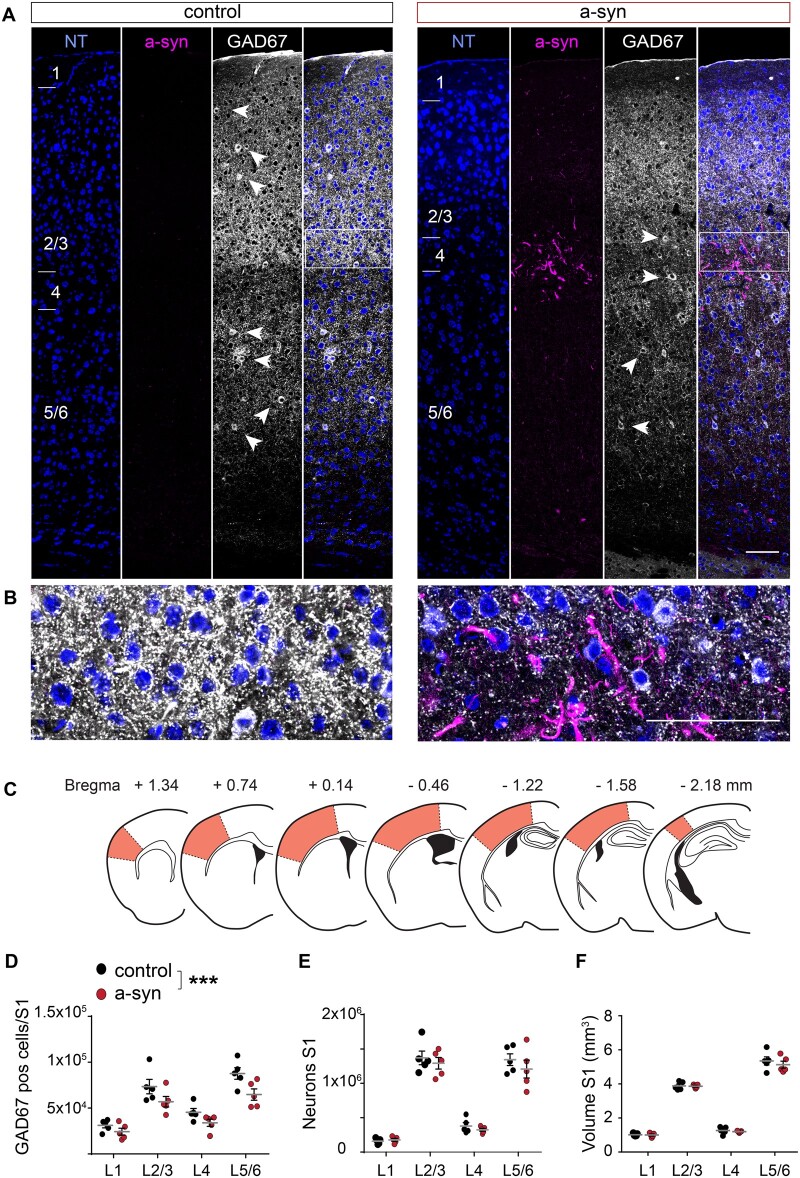
**Stereology of somatosensory cortex reveals reduction of inhibitory neurons.** (**A**) Representative examples of immunohistochemical stainings for neurotrace (NT, blue), phospho-synuclein (a-syn, magenta) and GAD67 (white arrow heads mark GAD67 positive GABAergic neurons) in S1 of a control and an a-syn PFF-seeded mouse assessed 9 months after striatal seeding. (**B**) Magnified area marked by a white box in (**A**). (**C**) Brain sections used for stereology to assess overall number of neurons and of GABAergic cells across all cortical layers in the entire S1 area (orange area). (**D**) The number of GAD67 positive interneurons was significantly reduced in a-syn mice [two-way ANOVA, effect of group *F*(1,32) = 14.69, *P* = 0.0006; effect of layer *F*(3,32) = 35.12, *P* < 0.0001; layer 5/6 *P* = 0.019; Bonferroni *post hoc* test, all other layers n.s], while neither (**E**) the total number of all neurons [effect of group *F*(1,32) = 1.41, *P* = 0.24, effect of layer *F*(3,32) = 132.2, *P* < 0.0001], nor (**F**) the cortical volume of S1 was affected [effect of group *F*(1,32) = 0.78, *P* = 0.39, effect of layer *F*(3,32) = 530.3, *P* < 0.0001]. Data are mean ± SEM in (**D, E and F**). Scale bar 100 µm in (**A and B**). *** *P* < 0.001.

As we observed a loss of dendritic spines on apical dendrites of pyramidal neurons in layer V in S1 upon striatal a-syn seeding in our previous work,^9^ we next asked whether a more global loss of synapses and/or neurons in S1 could be triggered by the a-syn spreading. To this end, we quantified the number of excitatory and inhibitory neurons by performing stereology and measured the synaptic density in the same area. While we neither observed a change in synaptic density (Supplementary Fig. 6), neuronal density or cortical volume (Fig. 4A–F), we found a reduction in the number of GAD67-positive inhibitory cells (Fig. 4D), which was most prominently seen in layer 5/6.

## Discussion

Cortical dysfunction is central to the development of the cognitive decline in a-synucleinopathies, such as Parkinson’s disease and LBD.^2–4^ However, the consequences of pathological a-syn on neuronal network function, particularly in the cerebral cortex, remain poorly understood. To address this question, we recorded neuronal activity in awake, behaving mice using two-photon calcium imaging 9 months after a single intrastriatal injection of a-syn PFFs. We and others have shown that striatal seeding of PFFs causes neuritic and somatic inclusions across all cortical layers in the absence of overt motor deficits.^18^^,^^19^ The observed a-syn spreading was accompanied by a loss of dendritic spines of layer 5 pyramidal neurons in S1, a region upstream of the inoculated striatum.^9^ We here now demonstrate that striatal a-syn seeding also results in neuronal dysfunction in the cortex. Using 2P *in vivo* imaging, we revealed a pronounced increase in neuronal activity in response to whisking (hyperreactivity) in a-syn PFF-injected mice. The observed elevated population response in S1 can be attributed to an increase in the fraction of whisking responsive cells and strongly suggests an altered excitation/inhibition balance. Furthermore, stronger pairwise neuronal activity correlations indicate increased network synchrony. Acute application of PFFs has recently been shown to cause spine loss and reduce neuronal activity.^8^ In contrast to acute effects, our data suggest that under chronic conditions, PFF seeding can cause the opposite effect on the network level. Mechanistically, hyperreactivity could be a consequence of the observed ∼25% reduction of GAD67-positive interneurons in S1 in a-syn PFF-seeded mice, occurring after the long incubation period of 9 months. This pronounced drop in GAD67 expression levels is likely causing an inhibitory deficit within the local microcircuitry. Additional mechanisms, for instance conveyed by activated glia cells, which we have shown earlier,^9^ are conceivable, too and need to be addressed in future studies. Importantly, the majority of cortical neurons, however, lack Lewy-neurite like aggregates both in our seeded mice and in humans and therefore are likely exposed to disease-mechanisms other than an overload of fibrillar a-syn. Earlier work conducted in a transgenic model that overexpresses human a-syn under the Thy1 promoter reported a dysregulation of Ca^2+^ dynamics *in vivo*, seen in a slower decay, which was suggested to be caused by compromised Ca^2+^ buffering.^5^ We here, however, did not observe any disturbances in the transient kinetics and thus conclude that in the PFF-seeding model calcium dynamics are not affected. However, the slow kinetics of genetically encoded calcium indicators, such as GCaMP6,^23^ may be insufficient to uncover smaller differences in the decay time. The two a-syn mouse models also strongly differ with respect to a-syn expression levels. While in our seeding model a-syn expression in cortex is mainly restricted to layer 4 and upper layer 5a, in the a-syn tg mouse model the majority of neurons is expressing large levels of a-syn.^5^ The prolonged state of progressive a-syn aggregation in a subset of neurons in our model more closely reflects the mostly sporadic human pathophysiology as opposed to transgenic mice overexpressing a-syn or cultured cells and tissues. Of note, hyperactivation of sensorimotor cortical areas was also shown in electrophysiological and functional MRI studies, conducted in parkinsonian rats and patients and have been attributed to both motor and psychotic symptoms in synucleinopathies.^24–26^ These data strongly emphasize the translational relevance of our findings also for human pathophysiology. Dysregulation of cortical activity in turn could lead to profound downstream changes in the striatum. Functional studies at cellular resolution are therefore crucial for defining the exact relationships in parkinsonian networks. Importantly, changes in neuronal excitability and/or activity, likely as a consequence of an excitation/inhibition imbalance, seem to evolve as a cardinal feature of neurodegenerative diseases and thus warrant further scrutiny to gain mechanistic insight and to explore its relevance for disease initiation and progression.^15^^,^^20^^,^^27–29^

Taken together, we here provide compelling evidence for a functional impact of seeded a-syn on cortical networks by overall increasing excitability, pointing towards a selective vulnerability of inhibitory interneurons. Importantly, our data demonstrate that chronic effects of a-syn accumulation differ from acute effects, causing an opposite impact at the network level through cell type-specific vulnerability, supporting the notion that spreading of a-syn alters the excitatory/inhibitory balance in cortical circuits.

## Supplementary material

Supplementary material is available at *Brain Communications* online.

## Ethics approval

All experiments were approved by the Bavarian government (Az. 55.2-1-54-2532-163-13).

## Supplementary Material

fcab273_Supplementary_Data

## Abbreviations

a-syn =: alpha-synuclein
Ca^2+^ =: calcium ions
ER =: endoplasmic reticulum
FOV =: field of view
GABA =: gamma-aminobutyric acid
GAD67 =: glutamic acid decarboxylase 67
KS =: Kolmogorov–Smirnov
LBD =: Lewy body dementia
PFF =: preformed fibrils
ROI =: region of interest
S1 =: primary somatosensory cortex
Tg =: transgenic
WT =: wild-type
