# Supplementary material for: Cortical circuit dysfunction in a mouse model of alpha-synucleinopathy in vivo
Source: Brain Commun. 2021 Nov 15;3(4):fcab273. doi: 10.1093/braincomms/fcab273 (PMC8643497; doi:10.1093/braincomms/fcab273)
Supplement: fcab273_Supplementary_Data [file fcab273_supplementary_data.doc]

**Supplementary Material**

Cortical circuit dysfunction in a mouse model of alpha-synucleinopathy *in vivo*

Sonja Blumenstock, PhD^1,2,3,4^, Fanfan Sun, PhD^1^, Caroline Klaus, M.Sc.^1^, Petar Marinković, PhD^1,5^, Carmelo Sgobio, PhD^1^, Lars Paeger, PhD^1^, Sabine Liebscher, MD, PhD^3,6,7^* and Jochen Herms, MD^1,2,3^*

^1^German Center for Neurodegenerative Diseases (DZNE), 81377 Munich, Germany
^2^Center for Neuropathology and Prion Research, Ludwig-Maximilians University Munich, 81377 Munich, Germany
^3^Munich Cluster for Systems Neurology (SyNergy), 81377 Munich, Germany
^4^Current Address: Molecular Neurodegeneration research group, Max Planck Institute of Neurobiology, 82152 Martinsried, Germany
^5^Current Address: Department of Pharmacy, Ludwig-Maximilians University, 81377 Munich, Germany
^6^Institute of Clinical Neuroimmunology, Klinikum der Universität München, Ludwig-Maximilians University, 82152 Martinsried, Germany
^7^Biomedical Center, Medical Faculty, Ludwig-Maximilians University Munich, 82152 Martinsried, Germany

* shared senior authorship

Correspondence: sabine.liebscher@med.uni-muenchen.de, jochen.herms@med.uni-muenchen.de

**Content:**

Suppl. Table 1

Suppl. Fig. S1. Striatal a-syn seeding triggers Lewy-like aggregate formation in the neocortex.

Suppl. Fig. S2. GCaMP6s is expressed primarily in excitatory neurons.

Suppl. Fig. S3. Neuronal activity is not altered during anesthesia.

Suppl. Fig. S4. Whisking behavior is not affected in a-syn mice.

Suppl. Fig. S5. Transient kinetics are not affected in a-syn mice.

Suppl. Fig. S6. Synapse density does not differ in a-syn and control mice.

**Suppl. Table 1.** Parameters for stereological cell counting in the cortex as well as the coefficient error and the average cell count per sampling site for each marker

| Ctx SSp | | Counting frame | Sampling  grid size | Coefficient of error (Gundersen), m=1 | Average cell counts  /sampling site |
| --- | --- | --- | --- | --- | --- |
| GAD67 | PBS | 100x100x8 µm | 300x300 µm | 0.12 | 1.10 |
|  | PFF | 100x100x8 µm | 300x300 µm | 0.13 | 0.84 |
| Neurotrace | PBS | 20 x 20 x12 µm | 200x200 µm | 0.11 | 1.22 |
|  | PFF | 20 x 20 x12 µm | 200x200 µm | 0.11 | 1.14 |


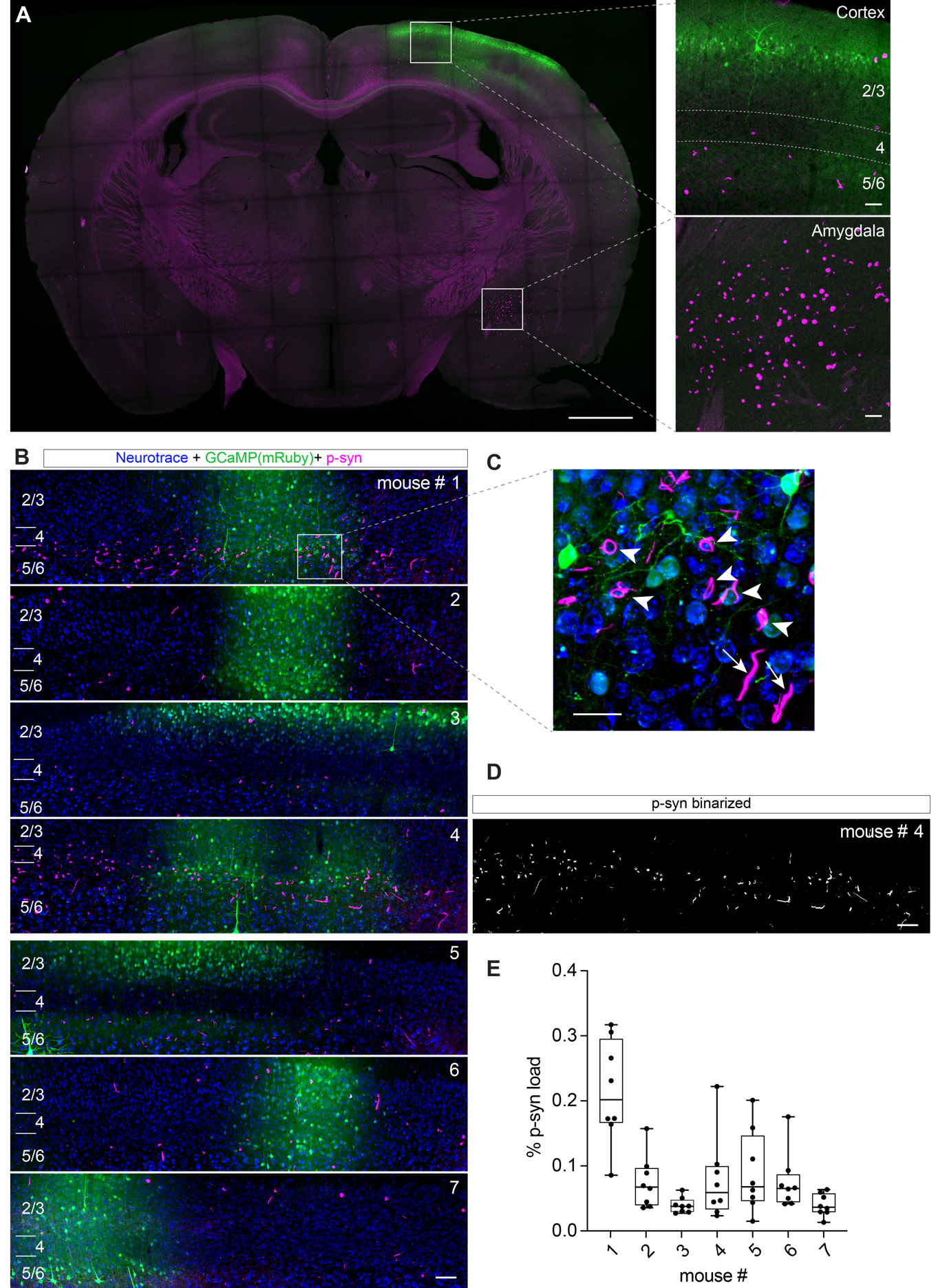


**Suppl. Fig. S1. Striatal a-syn seeding triggers Lewy-like aggregate formation in the neocortex.
(A)** Coronal section of a brain used for *in vivo* imaging showing the typical spread of a-syn aggregates (p-syn (magenta), GCaMP6 (green)). **(B)** Overview of S1 from all *in vivo* imaged mice. **(C)** Both neuritic (arrows) and mature somatic (arrow heads) a-syn inclusions are present. **(D)** Binarized a-syn signal. **(E)** Quantification of % area covered by a-syn aggregates in S1. Scale bars: A: 1 mm (left), 100 µm (right); B, D: 50 µm; C: 20 µm.


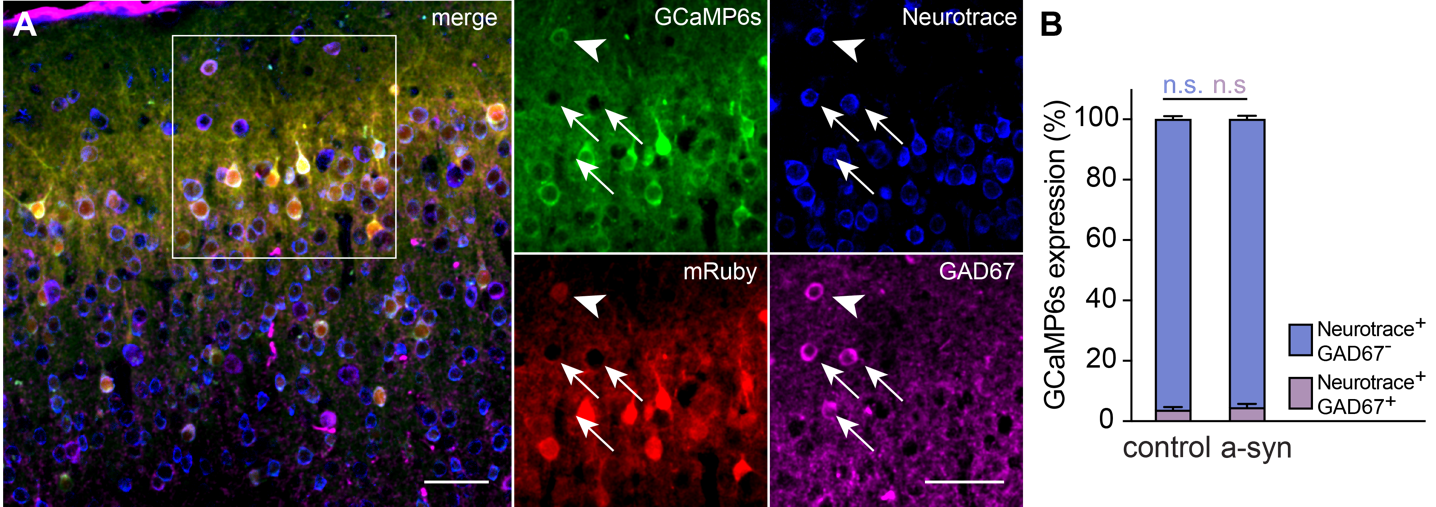


**Suppl. Fig. S2. GCaMP6s is expressed primarily in excitatory neurons.**

**(A)** Staining of inhibitory interneurons (GAD67) and a neuronal marker (Neurotrace) of the *in vivo* imaged area. Arrows mark GAD67 positive interneurons negative for GCaMP6s, arrowhead marks a neuron double positive for GCaMP6s and GAD67. **(B)** The fraction of interneurons out of all GCaMP6s expressing neurons accounts for 3.6 ± 1.39% (control) and 4.8 ± 1.57% (a-syn) cells. Two-way ANOVA with Bonferroni’s multiple comparison test. n.s. not significant. Scale bar in A: 50 µm.


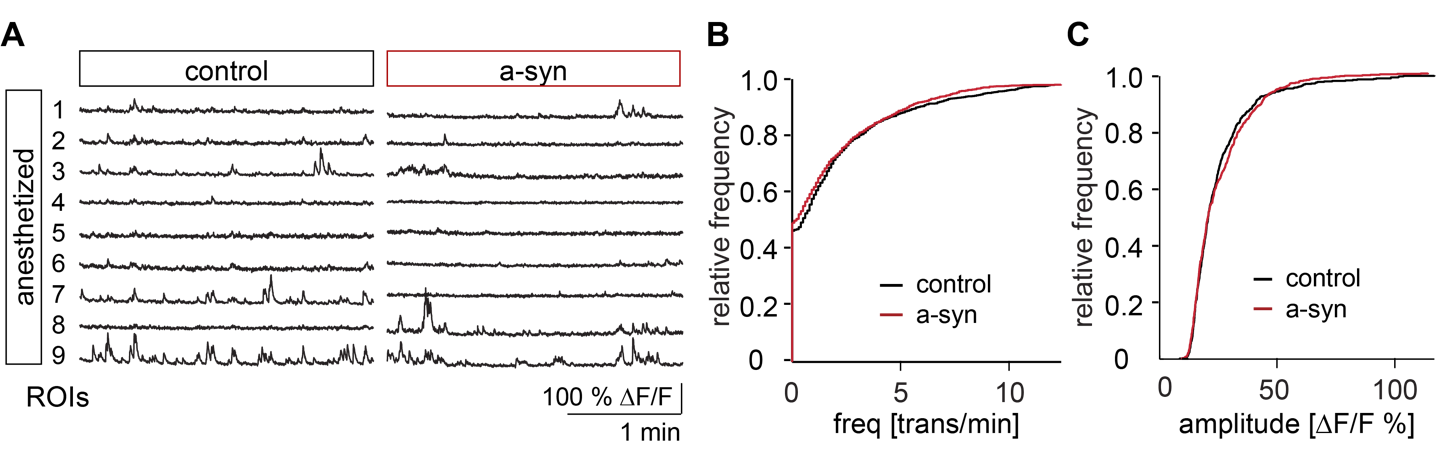


**Suppl. Fig. S3: Neuronal activity is not altered during anesthesia.**

**(A)** Examples of calcium traces from regions of interest (ROIs) in control and a-syn mice acquired under anesthesia. **(B)** The distribution of transient frequencies (P = 0.15, KS test, control n = 1561 ROIs, a-syn n = 1534 ROIs) **(C)** and transient amplitudes of active neurons (P = 0.22, KS test, control n = 659 ROIs, a-syn n = 566 ROIs) did not differ between control and a-syn mice for neuronal activity recorded under anesthesia.


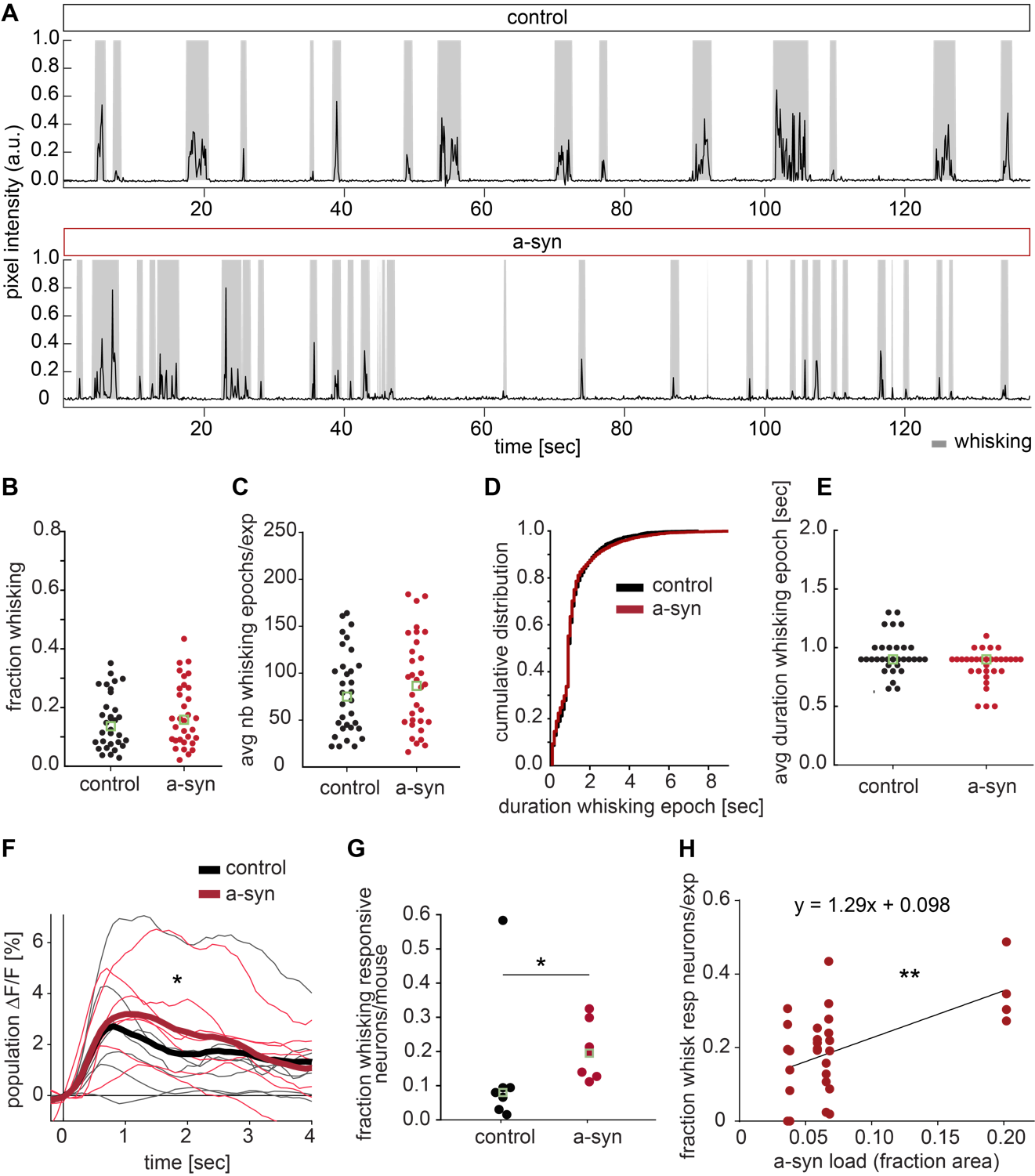


**Suppl. Fig. S4: Whisking behavior is not affected in a-syn mice.**

**(A)** Representative example of whisking raw data in control and a-syn mice, obtained from pixel intensity measurements of the whisker area (gray area denotes whisking epochs). **(B)** The time spent whisking was not different between control and a-syn mice (P = 0.45, ranksum test). **(C)** The number of whisking epochs per experiments did not differ (P = 0.485, ranksum test), **(D)** neither did the distribution of the duration of individual whisking epochs (P = 0.065, KS test), **(E)** nor the average whisking duration per experiment (P = 0.057, ranksum test). **(F)** The population response to whisking of individual mice was higher in a-syn mice compared to control (control n = 7, a-syn n = 7 mice, P < 0.05, bootstrapped CI of difference in average response 1-2 sec after whisking onset). **(G)** The fraction of whisking responsive mice was significantly higher in a-syn seeded animals (P = 0.026, ranksum test, control n = 7, a-syn n = 7 mice). **(H)** The fraction of whisking responsive neurons was positively correlated with the load of a-syn fibrils (P = 0.0017, Pearson’s R = 0.54, control n = 32, a-syn n = 32 experiments). Data are individual experiments (B,C,E), averages of mice (F,G,H, overlaid by the median - green square) or individual ROIs (D). * P < 0.05, ** P < 0.01.


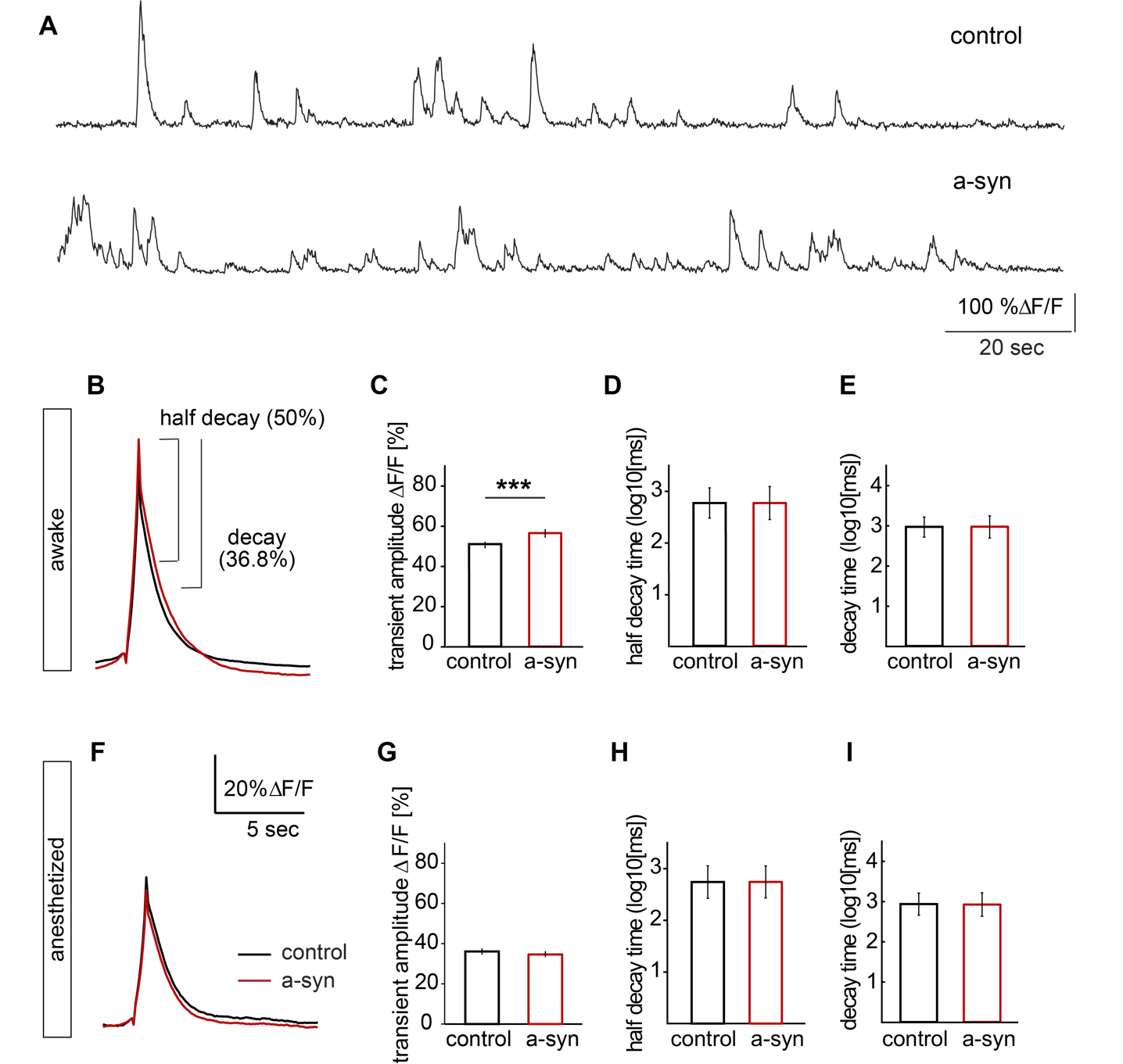


**Suppl. Fig. S5. Transient kinetics are not affected in a-syn mice.**

**(A)** Example of a calcium trace from a control and a-syn mice acquired during wakefulness. **(B)** Mean calcium transient of all ROIs in control and a-syn mice during wakefulness. **(C)** Average amplitudes of active neurons during wakefulness (P < 10-6, ranksum test) **(D)** The half decay time (P = 0.95, student’s t-test) and **(E)** decay time (P = 0.65, student’s t-test) did not differ between control and a-syn mice. **(F)** Mean calcium transient of all ROIs in control and a-syn mice under anesthesia. **(G)** Average amplitudes of active neurons under anesthesia (P = 0.055, ranksum test) **(H)** The half decay time (P = 0.92, student’s t-test) and (I) decay time (P = 0.44, student’s t-test) did not differ between control and a-syn mice. Data are median ± CI (C,G), mean ± SD (D,E, H,I). *** P < 0.001.

**
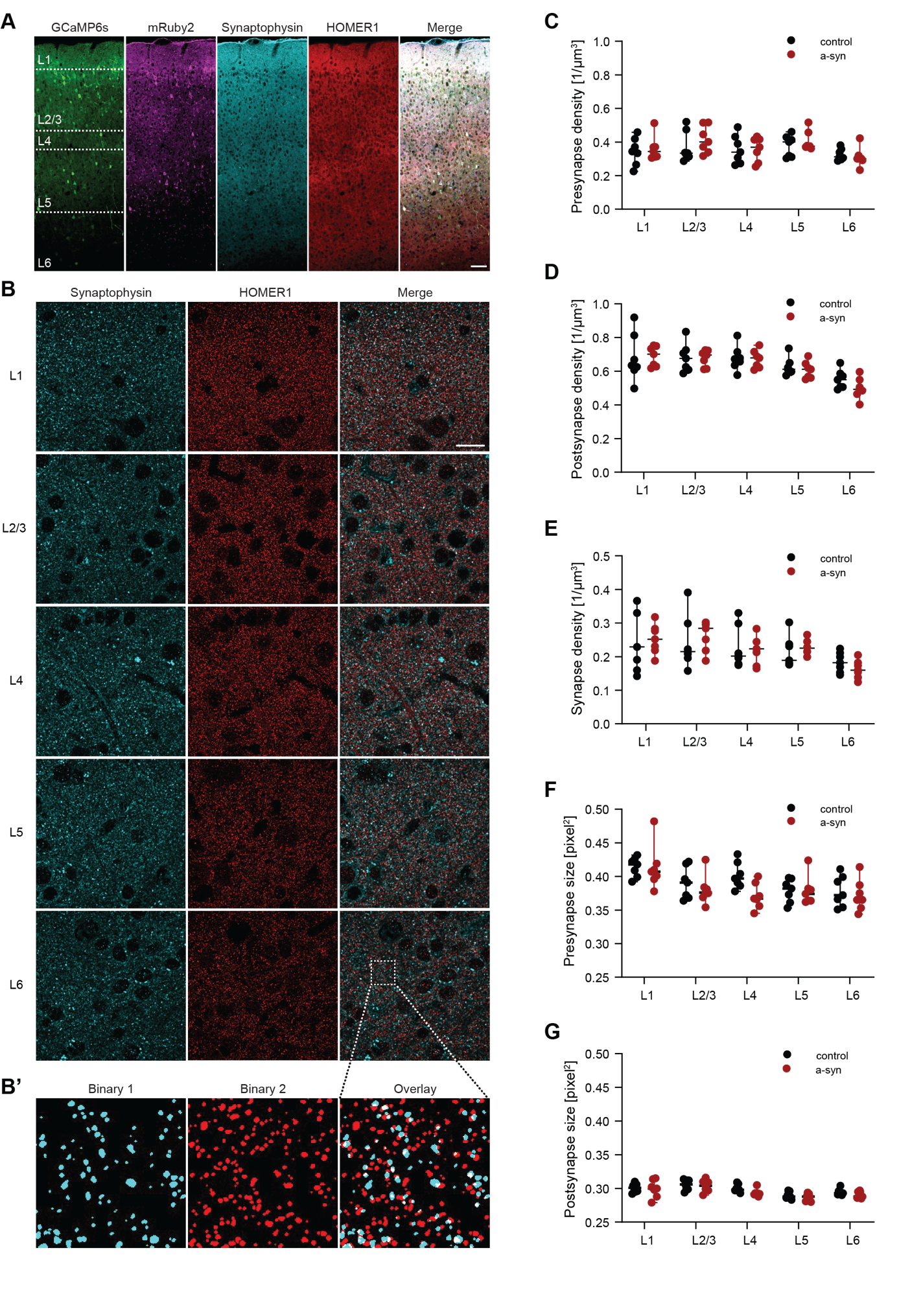
**

**Suppl. Fig. S6: Synapse density does not differ in a-syn and control mice.**

**(A)** Tile image of S1 cortical layers at virus injection side, showing GCaMP6s/ mRuby2 expression and pre-/postsynaptic immunosignal (scale bar: 100 µm). **(B)** Presynaptic (Synaptophysin) and postsynaptic (HOMER1) immunosignal across cortical layers. In the merged image, colocalized signals were identified as synapses (scale bar: 20 µm) **(B’)** Binary image of pre-/postsynaptic puncta used for quantifying colocalized signals. **(C)** Presynaptic, **(D)** postsynaptic and **(E)** total synaptic density separated in cortical layers, do not show a difference between a-syn PFF seeded and control animals (n = 7 animals per group). **(F / G)** Size of pre- and postsynaptic puncta. Data are expressed as median +/- 95% confidence interval. Significance between groups was analyzed by two-way ANOVA test. Example pictures in A and B are taken from a seeded animal.
